# Feather Corticosterone Measurements of Greater Flamingos Living under Different Forms of Flight Restraint

**DOI:** 10.3390/ani10040605

**Published:** 2020-04-01

**Authors:** Lukas Reese, Katrin Baumgartner, Lorenzo von Fersen, Roswitha Merle, Mechthild Ladwig-Wiegard, Hermann Will, Gudrun Haase, Oriol Tallo-Parra, Annaïs Carbajal, Manel Lopez-Bejar, Christa Thöne-Reineke

**Affiliations:** 1 Animal Behaviour and Laboratory Animal Science, Institute of Animal Welfare, Freie Universität Berlin, Königsweg 67, D-14163 Berlin, Germany; Mechthild.Ladwig-Wiegard@fu-berlin.de (M.L.-W.); zootieraerztin@vogelpark-marlow.de (G.H.); Christa.Thoene-Reineke@fu-berlin.de (C.T.-R.); 2 Tiergarten Nürnberg, Am Tiergarten 30, D-90480 Nuremberg, Germany; Katrin.Baumgartner@stadt.nuernberg.de (K.B.); lvfersen@odn.de (L.v.F.); Hermann.Will@stadt.nuernberg.de (H.W.); 3 Institute for Veterinary Epidemiology and Biostatistics, Freie Universität Berlin, Königsweg 67, D-14163 Berlin, Germany; Roswitha.Merle@fu-berlin.de; 4 Veterinary Faculty, Universitat Autònoma de Barcelona, Campus UAB, 08193 Bellaterra, Spain; Oriol.Tallo@uab.cat (O.T.-P.); anais.carbajal@uab.cat (A.C.); Manel.Lopez.Bejar@uab.cat (M.L.-B.); 5 College of Veterinary medicine, Western University of Health Sciences, Pomona, CA 91766, USA

**Keywords:** feather corticosterone, animal welfare, Greater Flamingo, deflighting, pinioning, bird

## Abstract

**Simple Summary:**

Greater Flamingos are commonly kept under flight restraint in zoos. Some are pinioned, others only feather clipped and some remain physically intact but live in aviaries that are often not large enough to fly. In this study, we compared these three groups by measuring corticosterone (a hormone associated with stress) in their feathers in order to find out which of the restraining methods is most compatible with animal welfare. Additionally, we carried out behavioral observations on all groups to detect potential stressors other than the status of flight itself. We expected to find differences in CORTf between deflighted and airworthy flamingos. However, no significant differences in feather corticosterone were measured between the three groups and the hypothesis was rejected. The most important factor for the level of corticosterone was found to be the zoological institution itself, reflecting the housing conditions. We hypothesize that the method by which a Greater Flamingo is hindered from flying does not have measurable effect on the corticosterone concentration in its feathers. Although these findings suggest that all methods are equally impacting animal welfare, we highlight the need for further improved studies based on this model.

**Abstract:**

Deflighting zoo birds is a practice that receives increasing criticism due to its presumed incompatibility with animal welfare. To our knowledge, this is the first approach to address this problem in a scientific way. To do this, we compared feather corticosterone (CORTf) from Greater Flamingos (*Phoenicopterus roseus*, n = 151) of different flight status (i.e., pinioned, feather clipped, airworthy) from twelve different zoological institutions. To complement the hormone measurements, behavioral observations (scan samplings) were conducted prior to feather sampling within the presumed time frame of feather growth. We hypothesized that CORTf of the deflighted flamingos would differ from CORTf of their airworthy conspecifics. No significant difference in CORTf was found between the three groups, and our hypothesis was rejected. However, the impact of the institution itself (i.e., the housing conditions) proved to be the most dominant variable (variance between the institutions = 53.82%). Due to high variability, the behavioral observations were evaluated descriptively but did not give rise to doubt the findings in CORTf. Therefore, we assume that the method of flight restraint of Greater Flamingos does not have a measurable effect on CORTf. We consider this model for evaluating animal welfare of zoo birds a useful tool and provide ideas for further adjustments for consecutive studies.

## 1. Introduction

The assessment of zoological animals’ well-being is a major challenge for everyone involved in the exhibiting and showcasing of wild animals. Particularly in terms of zoological birds, little has been published to enable a science-based welfare evaluation [1,2]. This also applies to the question of the behavioral significance of flight for zoological bird species. In Germany, however, a more stringent interpretation of §6 Animal Welfare Act has resulted not only in a strict prohibition of all irreversible deflighting procedures, but also in an inconsistent prosecution of wing clipping (i.e., feather clipping) depending on the local authorities [2,3,4]. Accordingly, the question of welfare implications of flight restraint in zoological kept birds has gained increased relevance in recent years; a change that does not only apply to Germany. In particular, pinioning (the amputation of the wing tip) has not only become a widely discussed topic amongst zoological representatives and zoological animal scientists but has also drawn the attention of politics and the general public [5,6,7,8,9]. Therefore, in some European countries (e.g., Austria, the Netherlands), irreversible deflighting techniques (i.e., pinioning and the extirpation or destruction of feather follicles) have been outlawed in order to meet the expectations of a supposed higher state of welfare [2,10]. However, there is no general consensus that a ban on deflighting actually serves this purpose [11]. Some authors state that under certain circumstances deflighting may provide a higher state of welfare than can be achieved without, at least for some species [12]. They argue that the commonly deflighted species are usually large birds that have a strong binding to the ground and/or water, for example flamingos (*Phoenicopteridae*), pelicans (*Pelicanidae*) and cranes (*Gruidae*), and that based on the behavioral knowledge about these species, flight can be considered a less important need. Therefore, they are commonly kept in open display on large areas, often in combination with large water surfaces, that offer the opportunity to show a diverse behavioral repertoire. Because of management limitations (e.g., preservation orders, statics, costs), a prohibition of deflighting procedures may result in smaller, less versatile aviaries that solely serve the purpose to keep intact birds, thereby compromising available space and structure without enabling actual flight [2,11].

It must be noted that both assumptions lack a scientific basis. It has neither been scientifically evaluated whether commonly deflighted bird species are in a lower state of welfare, nor if the ability to fly can be considered negligible or even outweighed by other, more important behaviors [2]. With a view to contribute to this question, we considered the Greater Flamingo (*Phoenicopterus roseus*) a suitable species for this study for the following reasons: (1) it is common and is the most frequently kept flamingo species in German zoos; (2) it is usually presented in larger flocks with more than ten individuals, which positively impacted the sample size; (3) it is commonly kept under flight restraint in open display [13]; (4) flamingos in general are relatively well-studied and belong to the most popular zoological birds [14] (thus a relatively large number of publications concerning biology and even welfare-related considerations of captive and free-ranging flamingos already exist and could be included in the evaluation [15,16,17,18,19,20,21]); and (5) flamingos are known to be powerful flyers that can travel long distances if needed [22]. On the other hand, observations from the wild suggest that they are reluctant flyers that only make use of their wings if absolutely necessary [19,23]. This is supported by observations from one of the few aviaries large enough to enable actual flight of flamingos, in Odense, Denmark [6], where Greater Flamingos are only rarely observed flying for short distances [2]. In their review about captive flamingo welfare, Rose et al. therefore suggest that flight-restraint might not affect the key behaviors that significantly impact on welfare [23]. However, they also highlight the need for scientific data in this context.

Glucocorticoids (GC) are well-described to play an important role in the response to stressful events in vertebrates. Along with an immediate enhanced secretion of catecholamines, the hypothalamic–pituitary–adrenal (HPA) axis in mammals and birds (hypothalamic–pituitary–interrenal (HPI) axis in reptiles, amphibians and fish) is activated, and within minutes a cascade of associated hormones and mediators (inter alia corticotropin-releasing hormone (CRH), adrenocorticotropin (ACTH), prolactin (PRL)) results in the release of GC into the bloodstream [24,25]. As a result, two waves of endocrine actions exist in a response to a stressor: a first adrenal catecholamine-mediated and a subsequent GC-mediated response, which are referred to as ‘fight-or-flight’ and ‘vigilance’ responses [24]. While the predominant GC in most mammals and fish is cortisol, in birds, reptiles, amphibia and rodents, it is corticosterone (CORT) [26,27]. The sudden increase in GC concentration leads to various effects in order to respond properly to threats and ensure survival. These include the increase of cardiac output and blood pressure, enhanced water retention, the metabolization of energy carriers (stimulation of gluconeogenesis, glycogenolysis, lipolysis and proteolysis) and protection against an overshooting immune response [24]. Additionally, CRH is believed to suppress hunger as well as reproductive physiology and behavior [24]. Via a negative feedback mechanism, sudden GC elevations are limited in time and their effects last from hours to days at the most [24]. The physiological consequences of GCs and their associated mediators are well-studied and have earned GCs the name ‘stress hormones’ [25].

The term ‘stress hormone’ is however considered misleading in modern endocrinology and is mostly avoided as it implicates a unidimensional view on the role of GC [25]. GCs are detectable not only at all times in vertebrates, but also in varying concentrations that cannot easily be related to stressors. Internal (e.g., sex, age, reproductive status, body condition) as well as external (e.g., season, climate) factors have shown to impact baseline GC concentrations [26]. Over the last two decades, various attempts have been made to better understand the role of these varying concentrations, especially in terms of how the HPA axis responds to long-lasting challenges, often referred to as ‘chronic stress’. Sapolsky et al. reviewed the physiological effects of GCs and highlighted their role in maintaining homeostasis throughout stressful events by categorizing their actions as permissive, suppressive, stimulating and preparative [24]. McEwen and Wingfield [28] proposed to apply the concept of allostasis [29] (stability, or homeostasis, through change) to the HPA axis, which has been picked up and modified by Romero et al. in the form of the ‘reactive scope model’ [30].

Measuring CORT concentrations in feathers (CORTf) is a rather new but increasingly used method in the field of stress research in birds [31]. Compared to the conventional measurement of plasma CORT, the assessment of CORTf has some advantages: (1) it is minimally or even noninvasive—feathers can be plucked both from live [32,33,34] or dead birds [35] or even collected from molting individuals without capturing them [31]; (2) it enables a retrospective, integrated measure of HPA-axis activity for the time period of feather growth and cannot be distorted by capture as long as feather growth is finished at the time of collection [35]; and (3) feathers are a stable matrix that apart from being stored dry and clean do not require additional storage conditions or processing [31,36,37,38,39]. These advantages are especially useful in wild animals and in field work when (repeated) access to birds is difficult and equipment is limited.

First described in 2008, Bartolotti et al. [32] showed not only that CORT is deposited into the growing feather, but also that its concentration increased when Red-legged Partridges (*Alectoris rufa*) experienced stress (in the form of a catch-and-release protocol) during feather growth. Consecutive studies provided evidence that the circulating CORT actually diffuses from the plasma into the feather and that plasma elevations also result in elevated CORTf [34,40,41]. Since then, numerous studies evaluated CORTf of different species, many of them focusing on carry-over effects of wild birds as a predictive measure for reproductive success [42,43], survival [36,44] or immunocompetence [45]. Only few controlled experiments have been conducted to evaluate the impact of environmental changes during feather growth on CORTf. However, results have consistently led to the conclusion that CORTf is a useful measure to show HPA-axis reactions to stimuli in order to predict an alteration in allostatic load. Fairhurst et al. [33] were able to show a short-term increase of CORTf followed by a long-term decrease in Clark’s Nutcrackers (*Nucifraga columbiana*) in response to enrichment. Will et al. [46] measured elevated CORTf levels in food-deprived Rhinoceros Auklet (*Cerorhinca moncerata*) chicks. Surprisingly, in a similar experiment, food-deprived Caspian Tern chicks (*Hydroprogne caspia*) were found to have lower CORTf levels than a normally fed control group, but the tern chicks also demonstrated reduced feather development which did not apply for the auklet chicks [47]. These results demonstrate that measurement of CORTf is a promising field that demands further research.

For interpretation of CORTf, knowledge about the feathers that are being used is essential. Molting patterns of Greater Flamingos are primarily described for the development from the immature to adult plumage [48,49] as well as for the remige molt [18,50]. In breeding populations, the molt of the flight feathers is dictated by the breeding season [50], which itself depends on season, local climates and food supplies in the habitat [18]. Breeding individuals usually molt their remiges after incubation, whereas nonbreeders usually molt during incubation. Shannon [50] observed that the cover feathers of different body parts of adult Caribbean flamingos were molted in a specific order over months beginning in the prebreeding season and extending into the period afterwards. Only the interscapular coverts appeared to be molted continuously throughout the whole year without showing any specific pattern. Changes in the feather coloration of chick-raising flamingos (feathers growing in this period are less colorful) indicate that body coverts are being molted at least twice a year [50].

Based on these findings, we assumed that CORTf of the interscapular coverts might reflect an average measure of the HPA axis of the previous six months. Our overall objective was to evaluate whether flamingos of different flight status showed differences in CORTf that allow conclusions to be drawn about their state of welfare. We hypothesized that if being deflighted goes along with severe confinements in the flamingos’ welfare, an increased allostatic load and consecutive changes in CORTf can be presumed. Therefore, we expected CORTf of these groups to be either higher or lower than CORTf of the airworthy conspecifics. To minimize the chance of misinterpretation, concomitant behavioral observations were carried out to detect additional potential influences on allostatic load other than the flight status.

## 2. Materials and Methods

### 2.1. Ethics

Plucking feathers is categorized as an ‘animal experiment’ according to German legislation. The experiment has been approved by the competent legal authorities of the respective German federal states where the participating zoos were located, since the experiment took place in 12 zoos in eight different federal states. The experimental design was first submitted to the District Government of Lower Franconia and approved in September 2016 (registered under the file number 55.2 DMS 2532-2-337). Based on this approval, the application was transferred to the remaining authorities and approved within the next months.

### 2.2. Zoological Institutions and Behavioral Observations

Twelve German zoological institutions with flocks of Greater Flamingos took part in the study. The inquiry to participate was sent to all members of the German Association of Zoological Gardens (Verband der Zoologischen Gärten e.V., VdZ) that kept Greater Flamingos at that time. The majority confirmed participation. Eleven zoos kept their flamingos in open display under flight restraint (i.e., pinioned and/or wing clipped), and one zoo (Zoo H) kept the flock in an aviary with most of the individuals therein having intact wings. Additionally, in one openly kept flock (Zoo D), single airworthy flamingos were among the deflighted majority (see Table 1).

Prior to feather sampling, all Greater Flamingo groups were visited between May and September 2016. Together with the responsible veterinarians, curators and keepers, information on the husbandry conditions such as group size, socialization, breeding status, demographic composition and flight status was collected by a questionnaire and categorized (see Table 1). Then, behavioral observations were carried out on three consecutive days in each institution. For the qualitative behavioral assessment, instantaneous scan sampling was used to characterize the proportionate behaviors within the flock [51]. The underlying ethogram was compiled after reviewing activity budgets from previous studies [52,53,54,55] and tested in a prerun series. The ethogram that was ultimately applied consisted of the following behaviors: foraging, resting, preening, locomotion (i.e., walking), reproductive behavior (including nesting, courtship display [56], feeding the young), aggression (taking in account the first two levels of aggression described by Schmitz and Baldassarre [57]), showing alarm and fluttering. The detailed ethogram can be found in Table S1.

Scans were recorded on three consecutive days in real time. Each day consisted of a morning session of two hours between 08:00 and 12:00 and a second two-hour-session in the afternoon between 13:00 and 17:00. These time frames of observation were chosen at least half an hour apart from daily disturbances involving zookeepers entering the exhibits (i.e., feeding, cleaning works, others). Behaviors were documented every 3 min on a tablet computer using the software Noldus (Pocket Observer, Wageningen, NL) and afterwards imported into Noldus (The Observer XT, Wageningen, NL). Scans always included the whole flock.

### 2.3. Feather Collection

Feather collection started in late autumn 2016 and went on until the end of the same year due to two reasons. First, the collected feathers were most likely to have grown within the time period of observation as well as the preceding spring and summer. This also represents the time of the year where all flamingos are living in their main exhibits; in some zoos they have to be kept indoors during winter due to cold climates. Second, for animal welfare reasons, the feather collection was done only during routine captures (i.e., routine medical examinations, wing clipping, transport to wintering quarters), and most of the German zoos carry out these procedures at the end of the year. Due to the highly variable time schedules of the individual zoos (on which the sampling depended on), the feather collection extended over a time frame of two months. For animal welfare reasons, only a representative number of individuals of each group was sampled depending on flock size, flight status and age. Greater Flamingos under one year of age were excluded. Feathers were plucked from the interscapular region of each individual [39,58] and stored dry and in the dark in labeled paper envelopes.

### 2.4. Corticosterone Extraction and Measurement

All feathers were inspected for integrity and cleanliness. The calamus was cut off and the feather length was individually measured up to an accuracy of 0.1 mm [38]. To homogenize feather samples between individuals, a total of at least 200 mm feather lengths was required. Due to different initial feather lengths, between two and ten feathers of the same type and length were selected from each sample, and the exact length and weight to the nearest 0.1 mg was recorded. The average length of actual samples was 293 ± 81 mm. For the CORTf extraction procedure, the protocol of Bartolotti et al. [32], modified and validated by Monclús et al. [36], was used. The feather samples were minced using a ball mill (Retsch, MM200 type) to obtain a feather particle size <2 mm. The feather dust of each sample was mixed with 1.5 mL methanol (99.9%) and put in a vortex (Vortex Mixer S0200-230 V-EU; Labnet International, NJ) for 30 min at room temperature. The mixture was then incubated for 18 h at 37 °C (G24 Environmental Incubation Shaker, New Brunswick Scientific, Edison, NJ, USA) and centrifuged at 6000 RPM (4000 × *g*) for 15 min (Hermle Z300K; Hermle Labortechnik, Wehingen, Germany). Afterwards, 0.75 mL of the supernatant were pipetted in a new sealable microtube and dried in an oven at 37 °C. The residue was reconstituted with 0.25 mL of the buffer solution provided by the used commercial enzyme immunoassay kit (ELISA Neogen Corporation, Ayr, UK), shaken for another minute using the vortex and frozen at −20 °C until analysis. The CORT measurement was performed as indicated by the manufacturer. All measured values were put in relation to the feather length as well as to its weight. For assay validation, samples were run in duplicates and triplicates. The inter- and intra-assay coefficients of variation for the analysis were 7.37% and 4.68%, respectively. After these values had been confirmed, samples were measured in single runs randomly and blindly distributed over a total of five EIA kits.

### 2.5. Statistical Analysis

Statistical analysis was performed using IBM SPSS Statistics v. 24. Prior to analysis, frequency tables were created and continuous values (CORTf) were checked for normal distribution by visual inspection and Shapiro–Wilk test. Since CORTf violated normality assumptions, logarithmic values were calculated (which were normally distributed) and used for further analyses.

Frequency tables were created for each institution, sorted and named (from A to L) by their mean CORTf value in ascending order and visualized in a box-and-whisker diagram. Additionally, for those institutions that kept airworthy flamingos together with deflighted animals (i.e., zoos D and H), a direct comparison within each population was made by using Student´s t-tests.

A linear mixed regression model was chosen to determine the influence of different variables on CORTf. Therefore, logCORTf was set as a dependent variable, the zoological institution as a random factor and the remaining variables as fixed factors. All variables that were not related to the behavioral observations (i.e., flight status, sex, socialization, age, breeding status, group size) and their two-way-interactions were included in the full model. Manual backward selection of variables was used to remove nonsignificant variables. The change in the **−**2 log-likelihood (**−**2 LL) was used as decision criterium. Secondly, the activity budgets from the behavioral observations were inserted one by one and inspected for their influence on the −2 LL. Thus, the final model included all variables that were statistically significant as well as those that had significantly affected the **−**2 LL. Residuals were checked for normality and homoscedasticity. All *p*-values below 0.05 were considered significant.

The data collected during behavioral observations were used to create activity budgets for each group. These are visualized in a bar diagram for qualitative analysis.

## 3. Results

Median CORTf of all samples (n = 151) regardless of their origin was 11.46 pg/mm (IQR: 5.9). The minimum was 2.66 pg/mm and the maximum was 20.93 pg/mm.

**Figure 1:**
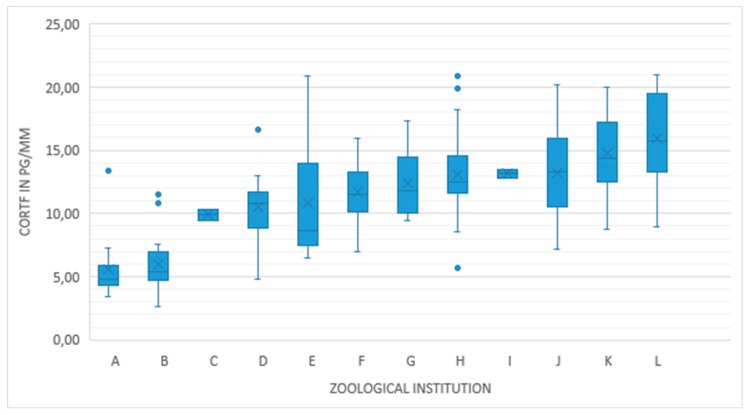
Feather corticosterone (CORTf) values of 151 Greater Flamingos within 12 zoological institutions.

Figure 1 shows the distribution of CORTf within the individual zoological institutions. In every individual zoo the CORTf values spread across their respective means, which range from 5.62 pg/mm for zoo A up to 15.96 pg/mm for zoo L.

Student´s t-tests for the zoos with flamingo groups that contained deflighted and airworthy individuals revealed no significant differences in logCORTf between the two groups of each population despite mean logCORTf values appearing to be slightly higher in airworthy flamingos than in their deflighted conspecifics in both populations. In Zoo D, mean CORTf was 11.11 ± 3.27 pg/mm (df = 4) for airworthy and 10.23 ± 2.41 pg/mm (df = 9) for pinioned flamingos (t = 0.594, *p* = 0.566); in Zoo H, mean CORTf was 13.37 ± 3.35 pg(mm (df = 27) for airworthy and 12.12 ± 1.71 pg/mm (df = 8) for deflighted (pinioned and feather-clipped) flamingos (t = 1.069, *p* = 0.293)

The initial linear mixed regression model included group size, breeding status, sex, age, status of flight and socialization. Since group size, age and breeding status of the colony were not found to have a significant effect on CORTf, they and the respective interactions were removed one by one. The final model included the variables flight status, sex and socialization as well as the interaction between socialization and sex . Influence of sex on CORTf was not statistically significant as a risk factor, nor was it significant when considered in interaction with socialization, but **−**2 LL changed significantly when removing one of the factors. In groups in interaction with other species (Socialization = 3), females tended to have higher CORTf values while males had higher values in groups with contact to other animals but without interaction (see Table 2).

The influence of the hierarchical level ‘zoological institution’ was investigated in terms of variance composition. It was found that 53.82% of the total variance was due to variance between zoological institutions (variance within the zoos: 0.0133; variance between the zoos: 0.0155). Residuals of the model were normally and homoscedastically distributed.

**Figure 2:**
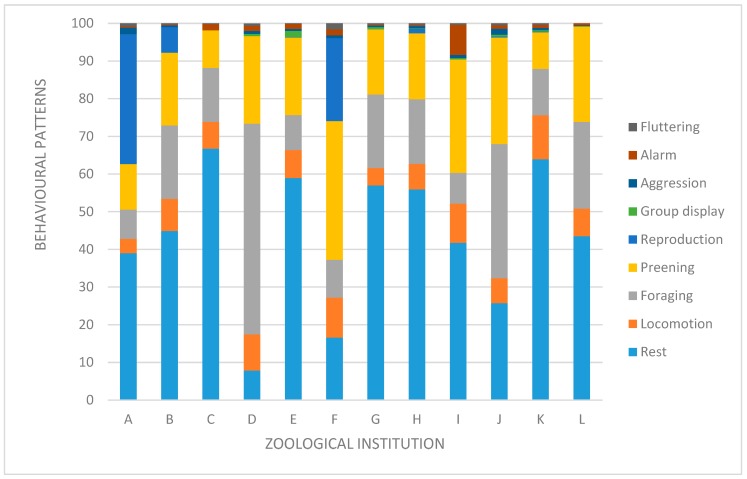
Activity budgets of the flocks of Greater Flamingos within the individual zoological institutions evaluated via scan sampling.

The activity budgets of the different groups are visualized in Figure 2.

The documented behavioral patterns show a high level of variability in their proportionate occurrence. This is impeded by a more variable breeding cycle: while in one zoo 50% of the group was involved in reproduction and their chicks hatched in May, in another zoo not more than 20% of the group were building nests in June, and others did not breed at all. Consecutively, incubating individuals that spent between 45% and 50% of the time per day on the nest were compared with parents feeding a few times per day or individuals that are not engaged in reproduction at all. Due to the low number of zoos and the high variability, we decided to do description only.

In some groups, observations could indicate an increased allostatic load. In zoo K, the Greater Flamingos shared their exhibit with a Black Crowned Crane *(Balearica pavonina),* and in zoo L they shared with a breeding pair of Common Cranes *(Grus grus)*. In both zoos, the cranes could be observed showing aggressive behavior repeatedly towards the flamingos (e.g., repressing, chasing, attacking). In zoo E, the flamingos shared their exhibit with a common crane, a pair of Demoiselle Cranes *(Grus virgo)*, White Storks *(Ciconia ciconia)* and a Great White Pelican *(Pelecanus onocrotalus)*. However, interspecific encounters during the time of observation did not result in more than neck-stretching towards the opponent and raising back and shoulder feathers, which are described as being a medium level of aggression also commonly witnessed as an intraspecific behavior.

In terms of actions linked to flying, no specific behaviors could be observed. None of the airworthy animals actually flew or tried to take off, including the deflighted flamingos.

## 4. Discussion

Significant differences in feather corticosterone were not found between any of the categorized groups (i.e., flight status, sex, group size, socialization, reproduction). The only variable found to have a strong impact on CORTf seemed to be the institution, reflecting differences in housing conditions. In the following, we discuss which conclusions can and, of equal importance, cannot be drawn.

The behavioral observations and activity budgets differed in various aspects. Therefore, these data could rarely be used for specific quantification of the deflighting technique on CORTf. Several husbandry conditions, e.g., feeding habits and times, group composition and habitat size, were not standardized but may have a huge impact on activity budgets, which interferes with the comparability. Additionally, the different groups were highly variable in terms of their breeding cycle and proportion. Although description is possible, statistical comparability in these cases is low. For further studies, behavioral observations should be improved.

In terms of qualitative analysis, the observations made during scan sampling were more promising. As predicted, in the two zoos in which cranes were observed to intentionally attack the flamingos (i.e., zoos K and L) the highest CORTf values were measured. This supports the hypothesis that repetitive and/or severe stressful events can have a measurable influence on CORTf [32,33].

One of the most surprising findings of this work were the large differences in CORTf between the institutions which proved to outweigh all other tested variables (e.g., sex, deflighting status). The median CORTf values were highly variable between the animal groups in different zoos (ranging between 4.77 ± 1.07 pg/mm and 15.75 ± 3.56 pg/mm), but they were relatively constant within the respective groups. This suggests that whatever external factors influence the HPA-axis activity in a group of Greater Flamingos appear to have a similar effect. For this, numerous influencing factors come into question and—although we tried to include as many as possible—some just prove not to be palpable or verifiable. For example, a Red Fox (*Vulpes vulpes)* pacing at the other side of the fence or an Eagle Owl (*Bubo bubo*) sitting in the flamingos’ visual field could be unnoticed nocturnal disturbances that still have the potential to significantly impact CORTf. Furthermore, even internal factors could play a role. For example, it has been shown that CORT baseline concentrations in Barn Owls (*Tyto alba*) are genetically correlated [59]. Therefore, population-dependent CORTf baseline concentrations are conceivable as well.

Another surprising finding was that the breeding status did not affect CORTf. For some bird species it has been shown that CORT elevates in prebreeding season [60] as well as during feeding of the chicks [61]—therefore, elevations in CORTf in breeding populations would have been predictable. It is possible that these findings do not apply to flamingos. However, we consider it more probable that these elevations were not detectable due to our study design, since as the flamingos were not marked individually, it was not possible to tell which individuals were actually involved in the breeding process. Additionally, for detection, the feathers should have grown in the exact time frames of elevated CORT.

For interpretation, it is important to note that we only compared potentially airworthy with deflighted animals. None of the birds included in this study had the opportunity to actually fly on a regular basis. The animals in the zoo D, although in an open display, stuck with their group, and those in zoo H were limited in flight due to the aviary’s dimensions. However, we consider this an important group as this is the case in most flamingo aviaries. For further studies it could be useful to examine samples from flamingos that live in exhibits large enough to allow actual flight, such as in Odense Zoo, Denmark [6]. Another valuable examination group might be free-ranging flamingos. As in any other exotic animal under human care, the wild animal in its natural habitat is supposed to be the reference for evaluating its behavioral needs. However, it must also be considered that free-ranging animals struggle with challenges that do not occur under human care and that might have an influence on their allostatic load, such as predation pressure, food shortage or extreme climates. These factors may complicate comparisons but are nevertheless certainly worth examining.

In our study, the status of flight did not have a significant impact on CORTf levels, neither in the regression model which included all individuals nor when comparing intact with deflighted flamingos from the same group. We therefore have to reject the hypothesis that CORTf from deflighted Greater Flamingos differs from CORTf of their intact conspecifics. Additionally, the method of deflighting did not have a measurable effect on allostatic load in our study. Whether this also applies to Greater Flamingos living in aviaries large enough to allow proper flight remains unclear. Caution is advised when drawing conclusions from these findings regarding the welfare status of the Greater Flamingos in this study. However, we detected highest CORTf levels in the two groups that were experiencing social stress (e.g., from being attacked), and therefore we assume that chronically stressful events do affect CORTf in Greater Flamingos. Consequently, it can be presumed that none of the techniques providing flight restraint mediate an effect associable to these conditions. At the same time, it also has to be considered that statistical noise in our study was quite high. Although we tried to take this into consideration by choosing the appropriate statistical model, it is likely that our model predominantly detected major alterations, whereas more subtle changes in CORTf due to one variable (in this case: status of flight) may remain below the limit of detection. This is aggravated by the fact that, by using interscapular feathers, only an average measure of the last six months can be provided, whereas it is not possible to determine exactly when the individual feathers were molted.

Additionally, the status of flight is a long-lasting (if not life-long) variable that might lead to a status of habituation or possibly even resignation. In Magellanic Penguins (*Spheniscus magellanicus*) [62], European Starlings (*Sturnus vulgaris*) [63] and Mallards (*Anas platyrhynchos*) [64], it has been shown that plasma corticosterone concentrations decreased over time in the presence of repetitive stressors (e.g., anthropogenic disturbances, catch-and-restraint protocols and a ‘work-out’). Therefore, it must be considered that Greater Flamingos may have become either accustomed to not being able to fly or even resigned.

## 5. Conclusions

We think that combining CORTf measurements and behavioral analysis is a promising approach, not only in terms of deflighting but also in general when evaluating bird welfare. However, further studies should improve the observation mode for reliable quantification. A valuable addition might be the inclusion of free-ranging animals to compare them with animals under human care. We also suggest prolonging the observation time to cover the whole day (eventually by extending the interval) and ideally even the nighttime as Greater Flamingos even show activity after dusk [20]. If possible, a time of the year with higher synchronicity between the different groups and their reproductive status should be chosen.

Our results indicate that the method of keeping a Greater Flamingo from flying might not affect CORTf levels. However, we see this work as a first approach but still sound a note of caution in terms of interpreting these results. Further studies with some adjustments discussed above are planned to confirm these results and to expand this model to other species affected by deflighting.

## Figures and Tables

**Table 1:** Overview of the involved zoological institutions and their flocks of Greater Flamingos.

Zoo	Total Group Size and Classification	Reproduction	Socialization	Sampled Animals + Flight Status
A	64 (II)	B	1	13 (2p + 11r)
B	67 (II)	B	2	19 (10p + 9r)
C	27 (I)	NB	2	9 (9p)
D	22 (I)	NB	1	15 (5a + 10p)
E	15 (I)	NB	3	15 (15p)
F	43 (II)	B	1	10 (10p)
G	2 (I)	NB	2	2 (2p)
H	46 (II)	B	1	37 (27a + 10p)
I	2 (I)	NB	2	2 (2p)
J	7 (I)	B	2	7 (4p + 3r)
K	36 (I)	NB	3	13 (3p + 10r)
L	12 (I)	NB	3	9 (9p)
**Total**				**151 (32a + 86p + 33r)**

I = 2–40; II ≥ 40; B = breeding group; NB = nonbreeding group; 1 = only flamingos (including all *Phoenicopteridae*); 2 = mixed-species exhibit, but no interaction witnessed; 3 = mixed-species exhibit, agonistic behavior witnessed; a = airworthy; p = pinioned/irreversibly deflighted; r = wing clipped.

**Table 2:** Final mixed linear regression model: investigation of 151 Greater Flamingos in 12 different zoological institutions with zoos as random effects and the influencing factors sex, status of flight, socialization as well as the interaction between sex and socialization as fixed effects. The global *p*-values for these variables are integrated in italics.

Influencing factor	Estimate logCORTf in pg/mm	*p*-Value	95% Confidence Interval	
			Lower Limit	Upper Limit
Intercept	1.067	<0.001	0.929	1.205
Female	0.090	0.200	0.015	0.166
Male	0	*0.257*	-	-
Airworthy	0.040	0.441	**−**0.063	0.113
Pinioned	**−**0.004	0.904	**−**0.077	0.068
Wing clipped	0	*0.502*	-	-
Socialization = 1	**−**0.133	0.176	**−**0.333	0.067
Socialization = 2	**−**0.086	0.350	**−**0.275	0.103
Socialization = 3	0	*0.136*	-	-
Sex = female * Socialization = 1	**−**0.090	0.075	**−**0.189	0.009
Sex = female * Socialization = 2	0.107	0.059	**−**0.217	0.004
ex = female * Socialization = 3	0	-	-	-
Sex = male * Socialization = 1	0	-	-	-
Sex = male * Socialization = 2	0	-	-	-
Sex = male * Socialization = 3	0	*0.109*	-	-

## Supplementary Materials

The following are available online at https://www.mdpi.com/2076-2615/10/4/605/s1, Table S1: Definition of the activity budget for Greater Flamingos.
