# Supplementary material for: Feather Corticosterone Measurements of Greater Flamingos Living under Different Forms of Flight Restraint
Source: Animals (Basel). 2020 Apr 1;10(4):605. doi: 10.3390/ani10040605 (PMC7222806; doi:10.3390/ani10040605)
Supplement: Supplementary file 1 [file animals-10-00605-s001.zip › Activity budgets.docx]

**Table S1:** **Definition of the activity budget for greater flamingos**

| Locomotion | Taking at least two steps on land if shown as primary behavior. If to be taken as secondary behavior like in agonistic or sexual actions or during foraging it is not to be counted. Occasionally shown preening during walking and swimming is included. |
| --- | --- |
| Resting | Includes the three usual resting/sleeping postures (lying on the ground, sitting on the metatarsal joints, standing on one or two legs) with the head below the wing as well as with the neck in a s-shaped form if not any other behavior is predominant (i.e. preening, social interaction) |
| Foraging | Includes all foraging methods, i.e. walking and filtering, up-ending, stamping, skimming as well as forceps-like feeding from buckets and drinking |
| Preening | Preening, stretching (includes Wing-Leg-Stretch if not shown as a group display), |
| Aggression | Agonistic behavior towards one animal (i.e. pecking)- only the active part is counted - or between two animals (i.e. male fights) |
| Fluttering | Fluttering of the wings, opening the wings due to windy conditions, hopping as well as actual flight attempts |
| Alarm | Any behavior that indicates a nervous inspection of the surrounding -> erect posture of the neck, eventually lifted beak and full awareness of the surrounding |
| Group display | Includes these ritualized displays at all times and even when done by one individual:   - Wing salute - Twist preen - Inverted wing salute - Wing-leg stretch - Head flagging   Only when following twist preen + false-feeding:   - Scratching   Only when done by at least two birds:   - Marching   Only when followed by head-flagging   - Alert posture |
| Reproduction | Behavior connected to reproduction, including nest-building, mating, incubating, feeding the young |
